# Distinct mechanisms govern recalibration to audio-visual discrepancies in remote and recent history

**DOI:** 10.1038/s41598-019-44984-9

**Published:** 2019-06-11

**Authors:** David M. Watson, Michael A. Akeroyd, Neil W. Roach, Ben S. Webb

**Affiliations:** 1 School of Psychology, University of Nottingham, Nottingham, NG7 2RD UK; 2 MRC Institute of Hearing Research, University of Nottingham, NG7 2RD Nottingham, UK; 3 Hearing Sciences, Division of Clinical Neuroscience, School of Medicine, University of Nottingham, Nottingham, NG7 2UH UK

**Keywords:** Auditory system, Perception, Sensory processing, Visual system, Human behaviour

## Abstract

To maintain perceptual coherence, the brain corrects for discrepancies between the senses. If, for example, lights are consistently offset from sounds, representations of auditory space are remapped to reduce this error (spatial recalibration). While recalibration effects have been observed following both brief and prolonged periods of adaptation, the relative contribution of discrepancies occurring over these timescales is unknown. Here we show that distinct multisensory recalibration mechanisms operate in remote and recent history. To characterise the dynamics of this spatial recalibration, we adapted human participants to audio-visual discrepancies for different durations, from 32 to 256 seconds, and measured the aftereffects on perceived auditory location. Recalibration effects saturated rapidly but decayed slowly, suggesting a combination of transient and sustained adaptation mechanisms. When long-term adaptation to an audio-visual discrepancy was immediately followed by a brief period of de-adaptation to an opposing discrepancy, recalibration was initially cancelled but subsequently reappeared with further testing. These dynamics were best fit by a multiple-exponential model that monitored audio-visual discrepancies over distinct timescales. Recent and remote recalibration mechanisms enable the brain to balance rapid adaptive changes to transient discrepancies that should be quickly forgotten against slower adaptive changes to persistent discrepancies likely to be more permanent.

## Introduction

To obtain a unified and precise percept in dynamic environments, the human brain integrates information across multiple sensory modalities. Perception of events and objects typically remains coherent despite discrepancies in the timing and/or spatial position of sensory signals between modalities^1^. To maintain perceptual binding of multisensory inputs, the brain corrects for errors arising between the senses. Consequently, multisensory discrepancies often lead to perceptual recalibration of corresponding modalities to minimize inter-modal errors. For example, changes in the perceived timing of multi-modal stimulus pairs have been reported following adaptation to temporally discrepant visuo-tactile^2–4^ and audio-visual^4–6^ stimuli. Similarly, repeated presentations of spatially discrepant visual and auditory stimuli lead to a perceptual recalibration of auditory space, such that perceived sound location is shifted to counteract the discrepancy – the “ventriloquism aftereffect” (VAE)^7–10^.

Over what timescale should the brain track multisensory discrepancies? In principle, systematic errors between senses could arise rapidly (e.g. transient changes in the environment) or persist over much longer epochs (e.g. gradual changes taking place over many years during childhood), requiring a perceptual system that can balance how it adapts over very different timescales. Multi-modal recalibration effects have been observed following a range of adapting periods. Early behavioural studies of both temporal^5,6^ and spatial^7,8,11–14^ audio-visual recalibration focussed on effects following upwards of several minutes of adaptation. However, more recent studies have further demonstrated rapid recalibration effects following just a few seconds or even a single trial of exposure to temporally^4,15^ or spatially^16–18^ discrepant audio-visual pairs. To maximise the benefit of multisensory recalibration and avoid spurious recalibration to sensory noise, the brain should match its rate of adaptation to the dynamics of the inter-modal error. However, if multiple sources of error exist, recalibration mechanisms should ideally be sensitive to the timescales over which such discrepancies occur^19^.

Here we use the ventriloquism aftereffect to distinguish whether control of timescales of multisensory recalibration is governed by a singular mechanism that grows in strength, or distinct mechanisms that gradually activate over time. Behavioural studies of unimodal sensory perception have supported the notion of distinct mechanisms by showing that several, potentially opposing, adaptation effects can be simultaneously maintained when they occur across different timescales, ranging from minutes to hours or even days^20–22^. However, the extent to which the same principles apply to multisensory perception remains unclear. Adaptation to audio-visual temporal offsets can yield radically different effects depending on the task and duration of adptation^23^. Similarly, a previous study of rapid spatial adaptation by Bruns and Röder demonstrated that the ventriloquism aftereffect shifts from being frequency-independent to frequency-dependent with increasing durations of adaptation, from a single trial up to four trials^17^. This suggests an adaptation process that is coupled to the timescales over which audio-visual discrepancies occur in the environment. However, these behavioural effects pertain to rapid adaptation to the very recent past, hence it remains unclear whether distinct or unitary recalibration mechanisms exist over longer timescales. A recent study by Bosen and colleagues showed the growth and decay of the VAE could be predicted either by a multiple-exponential model or a power model^24^ – yet it still remains unclear how these effects vary across different timescales, and whether opposing effects can be simultaneously maintained.

We measured the growth and decay of the ventriloquism aftereffect in the recent and remote past by adapting human participants to audio-visual spatial offsets for a range of durations (from 32 seconds to 256 seconds). To distinguish recalibration mechanism(s) operating at single or distinct timescales we adapted and de-adapted participants to equal and opposite spatial offsets for long and short durations respectively. If recalibration is controlled by a single mechanism that envelopes different timescales, initial perceptual aftereffects will be proportional to the combined effects of adaptor and de-adaptor, and decay back to baseline. If recalibration is controlled by distinct mechanisms operating at different timescales, aftereffects will initially be cancelled by the de-adaptation, but will subsequently reappear with further testing^21,25^.

## Results

### Experiment 1: magnitude of spatial recalibration

**Figure 1:**
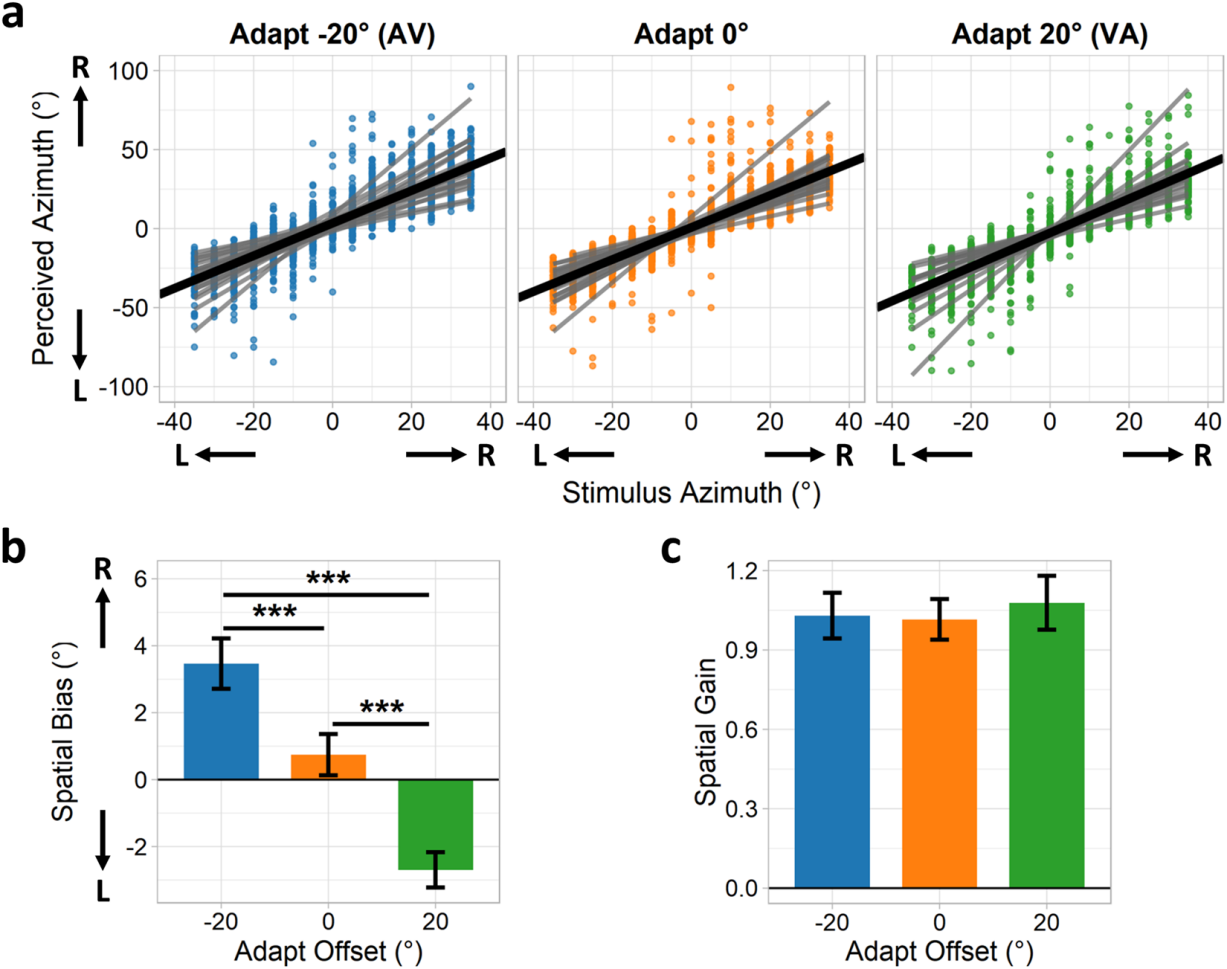
Experiment 1 results. (**a**) Participants’ perceived stimulus azimuth plotted against actual stimulus azimuth, following 1 minute of adaptation to audio-visual pairs spatially offset by −20° (audio left of visual), 0°, or 20° (audio right of visual). Data were concatenated across participants and entered into a series of mixed-effects linear regression analyses: thick black lines indicate average fixed-effects fits, thin grey lines indicate per-participant mixed-effects fits. Bar plots show (**b**) spatial bias and (**c**) spatial gain parameters, given by the fixed-effects intercept and slope coefficients respectively. Error bars indicate standard errors of the regression coefficients. Annotations indicate the results of pairwise t-tests contrasting the coefficients between adaptation offset conditions (***p < 0.001).

In an initial experiment we first sought to replicate the basic ventriloquism aftereffect (VAE) with our experimental paradigm. Visual stimuli (2D Gaussian blobs) were projected onto a large semi-circular screen that wrapped 180° around the participant, whilst auditory stimuli (pink noise bursts) were delivered over headphones with stimulus azimuth simulated via head-related transfer functions (HRTFs). Participants adapted for 1 minute to audio-visual stimulus pairs presented in a randomised order across 15 locations between −35° (left) and 35° (right) azimuth in 5° increments. Pairs either had a spatial discrepancy of −20° (audio left of visual), 0°, or 20° (audio right of visual) between them, counterbalanced across blocks. Stimulus duration was 500 ms, with a 300 ms interstimulus interval. Each audio-visual pair was presented five times in a row at a given location to facilitate allocating spatial attention, with one full pass over all 15 locations being completed over the 1-minute period. Participants were then tested on their ability to reproduce the azimuth of unimodal auditory stimuli presented within the same range of azimuths. Figure 1a shows participants’ perceived stimulus azimuth plotted against the actual stimulus azimuth for each adaptation condition. A clear, positive linear trend is evident in all conditions, indicating that the HRTFs were able to simulate stimulus azimuth effectively.

Data for each adaptation condition were entered into a series of mixed-effects linear regression analyses, with perceived and actual stimulus azimuth defined as the outcome and predictor variables respectively (Fig. 1a). Regression intercept coefficients represent participants’ spatial bias (Fig. 1b) and slope coefficients represent spatial gain (Fig. 1c). Adapting to spatially discrepant audio-visual pairs led to a shift in spatial bias in the direction of the visual offset. For instance, adapting to a −20° offset with the audio to the left of the visual led to a positive (rightward) shift in spatial bias, whilst adapting to a 20° offset with the audio to the right of the visual led to a negative (leftward) shift in spatial bias. Spatial gain parameters all appeared close to 1 and did not differ substantially across adaptation conditions.

For each parameter (spatial bias, spatial gain), mixed-effects coefficients across participants were entered into a one-way repeated-measures ANOVA with a main factor of adaptation offset (−20°, 0°, 20°). Greenhouse-Geisser corrections were applied as the data failed to meet the assumption of sphericity. For the spatial bias parameter, there was a significant main effect of adaptation offset (F(1.40, 26.63) = 69.67, *p* < 0.001, η^2^ = 0.49). Post-hoc paired-samples t-tests revealed significant differences in spatial bias between all pairwise combinations of adaptation offsets (−20° > 0°: t(19) = 7.81, *p* < 0.001, Hedges’ *g*_*av*_ = 0.92; 0° > 20°: t(19) = 6.64, *p* < 0.001, Hedges’ *g*_*av*_ = 1.42; −20° > 20°: t(19) = 9.38, *p* < 0.001, Hedges’ *g*_*av*_ = 2.24). There was no main effect of adaptation offset on the spatial gain parameters (F(1.55, 29.38) = 2.14, *p* = 0.131, η^2^ < 0.01).

Thus, adapting to an audio-visual spatial offset led to a perceptual recalibration of auditory space, shifting the perceived location of auditory stimuli in the direction of the visual offset. This confirmed that the basic VAE could be replicated with our experimental paradigm.

### Experiment 2: timescales of spatial recalibration

In a second experiment we aimed to test how the VAE grows and decays over time. Participants again adapted to audio-visual pairs with either −20°, 0°, or 20° offset, but we now varied the length of the adaptation period between 32 s, 64 s, 128 s, and 256 s. Stimulus details are the same as for experiment 1, except that during adaptation stimuli were presented across 8 locations between −35° and +35° azimuth in 10° increments. One, two, four, and eight passes over all locations were completed for each adaptation duration respectively.

**Figure 2:**
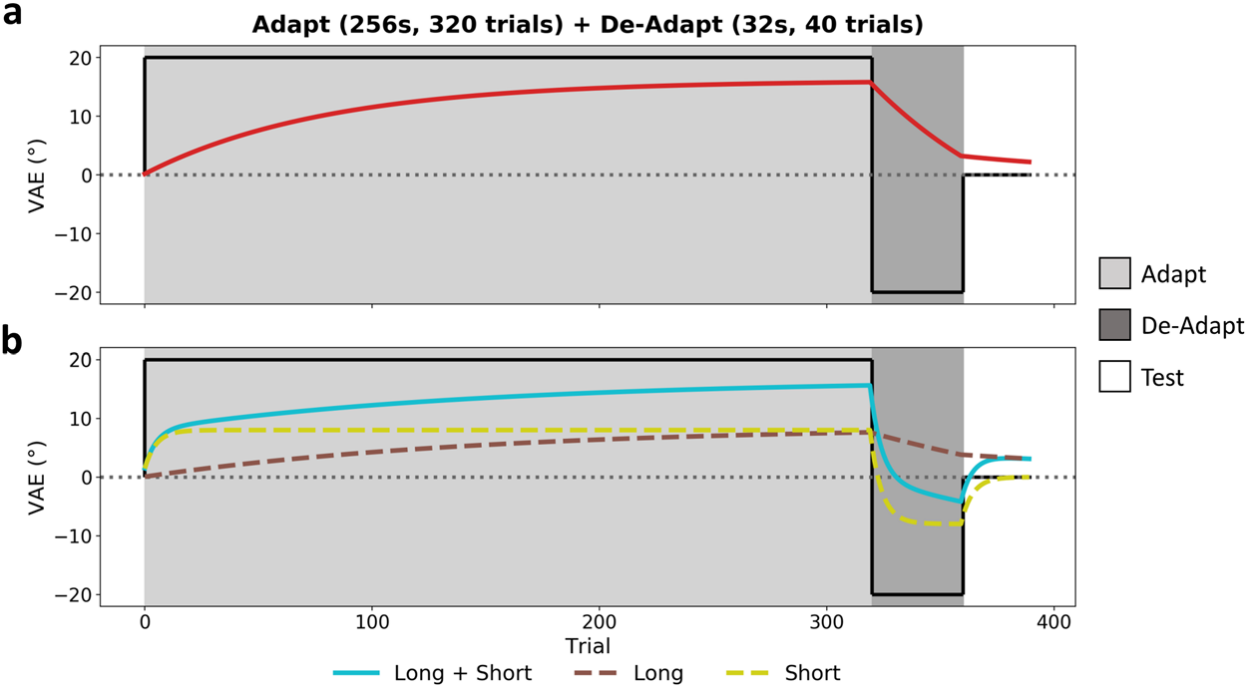
Model predictions for adapt/de-adapt condition VAEs, based on the outputs of exponential (leaky integrator) mechanisms. (**a**) Single-mechanism prediction. De-adaptor reduces adaptation initially built up by adaptor; during the test phase the VAE may be reduced, cancelled, or inverted, but in all cases will decay monotonically towards baseline over time. (**b**) Distinct-mechanisms prediction. A short-term mechanism will be mostly driven by the more recent de-adaptor at the start of the test phase, yielding a negative response that decays quickly up towards baseline. A long-term mechanism will be mostly driven by the longer duration adaptor, yielding a more sustained positive response that decays slowly down towards baseline. The net output of both mechanisms initially yields a reduced aftereffect as the mechanisms cancel, but there is a later positive recovery of the effect as the short-term mechanism decays whilst the long-term mechanism sustains.

In addition, we included an adapt/de-adapt condition in which participants first adapted to a given spatial offset (−20° or 20°) for 256 s and then immediately de-adapted to the opposing offset for 32 s. This allowed us to test whether it is possible to simultaneously maintain two opposing VAEs if they occur across different timescales (Fig. 2). Under a single-mechanism model, the de-adaptor simply reduces the adaptation built up by the initial adaptor. This may result in a reduced, cancelled, or even inverted aftereffect, but in all cases any effects will simply decay monotonically towards baseline. Under a distinct-mechanisms model, a short-term mechanism most sensitive to the immediate past would be mostly driven by the more recent de-adaptor at the start of the test phase, yielding a negative response that decays quickly towards baseline. Meanwhile, a long-term mechanism integrating information over wider time periods would be mostly driven by the initial longer duration adaptor, yielding a more sustained positive response. The net output of both mechanisms would initially produce a reduced aftereffect as the mechanisms cancel, followed by a later recovery in the direction predicted by the initial adaptor as the short-term mechanism decays whilst the long-term mechanism continues to sustain.

**Figure 3:**
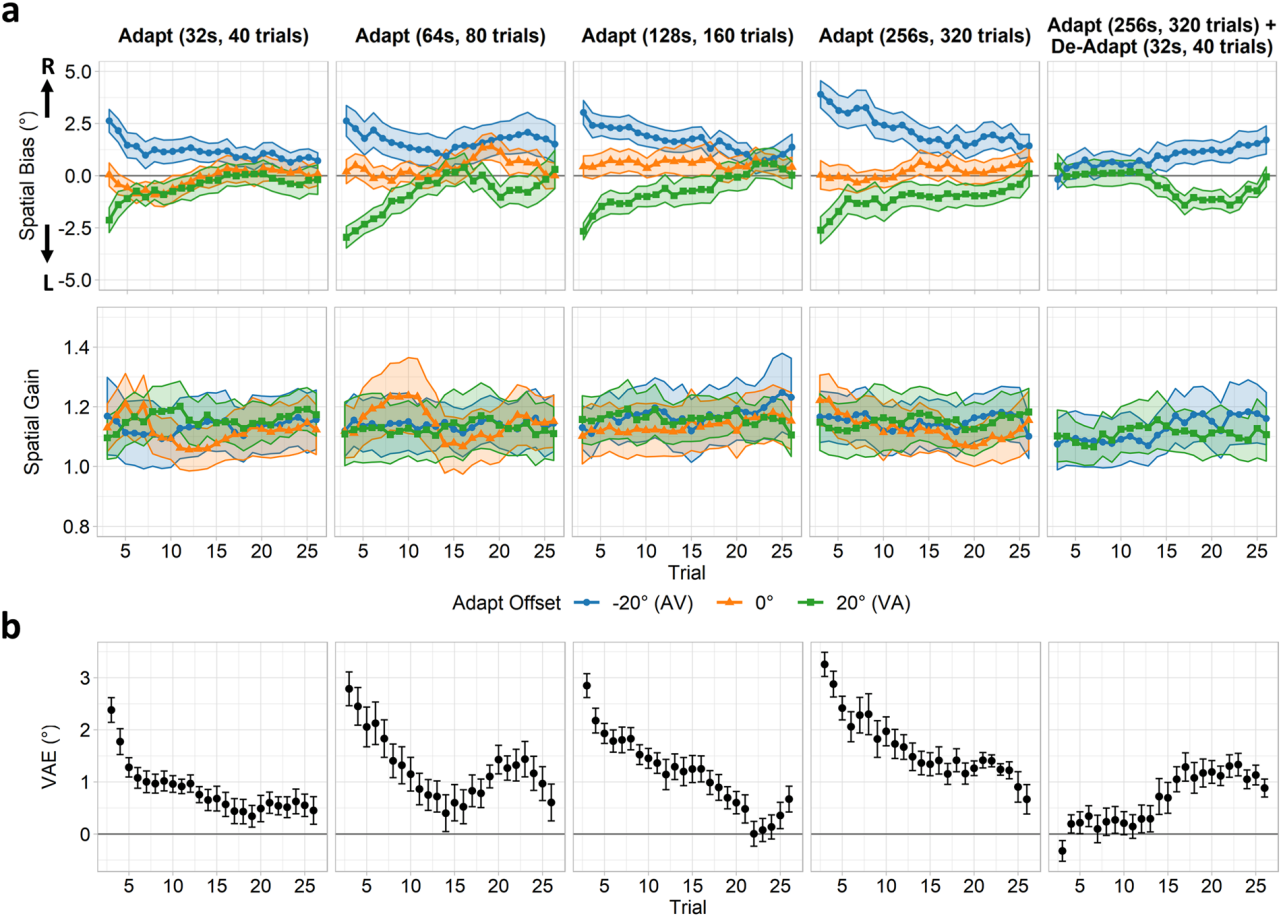
Results of mixed-effects linear regression analyses for Experiment 2, plotted over trials and differing durations of adaptation. Data were analysed using a sliding-window (7 trials, incremented in 1 trial steps); values are plotted against the middle trial of each window. (**a**) Spatial bias (intercept) and spatial gain (slope) coefficients are shown on the top and bottom rows respectively, following adaptation to audio-visual pairs spatially offset by −20° (blue; audio left of visual), 0° (orange), or 20° (green; audio right of visual). Shaded ribbons indicate standard errors of the regression coefficients. (**b**) VAE magnitudes, defined as the trial-wise average of −20° > 0° and 0° > 20° adaptation offset contrasts of the spatial bias coefficients. Error bars indicate the standard error of the mean across subjects.

Data from each test period were analysed using a sliding-trial window comprising 7 trials and incremented in 1 trial intervals. For each window, data were entered into separate mixed-effects linear regression analyses for each condition. The resulting spatial bias (intercept) and spatial gain (slope) coefficients are shown in Fig. 3a; coefficients are plotted against the middle trial of each window. We quantified the magnitude of the VAE by taking the trial-wise average of the −20° > 0° and 0° > 20° adaptation offset contrasts of the spatial bias coefficients (Fig. 3b). In the standard adaptation conditions, repeated exposure to spatial offsets caused shifts in spatial bias in the direction of the visual offset, indicating the presence of a VAE. These effects decayed in later trial windows as the effect of the adaptor deteriorated. The overall magnitude of the VAE did not increase substantially with increasing adaptation durations, suggesting a relatively fast acting adapting mechanism that saturated quickly. At the same time, the aftereffect often failed to fully decay to zero within the testing period, instead settling at a non-zero asymptotic level in later trials, suggestive of an additional slower acting mechanism predicting a more sustained response. By contrast, in the adapt/de-adapt condition the early trial windows showed little evidence of a shift in spatial bias, as the opposing VAEs caused by adaptors and de-adaptors cancelled. Crucially, however, a VAE was seen to re-emerge in later trial windows in the direction of the initial adapting offset. This indicates that the effects of the more recent but shorter-term de-adaptor had decayed, whilst the effects of the earlier but longer-term adaptor sustained. This demonstrates that distinct and opposing VAEs occurring across different timescales could be simultaneously maintained. Meanwhile, spatial gain coefficients appeared close to 1 in all conditions and did not differ reliably across either adaptation offsets or durations.

**Figure 4:**
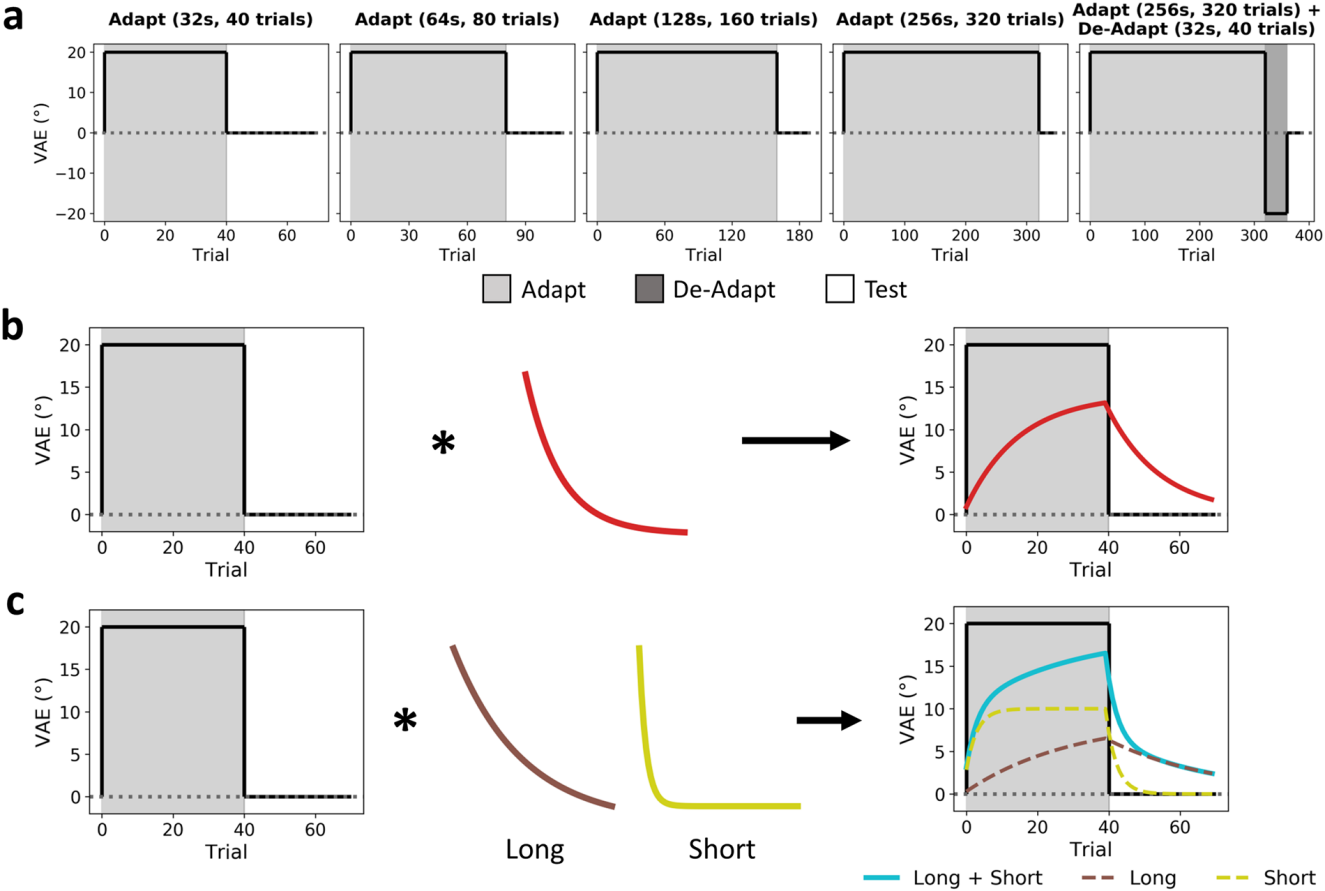
Schematic illustration of modelling procedure. (**a**) A series of boxcar models simulate the magnitude of the spatial offset during adaptation, de-adaptation, and test periods. (**b**,**c**) Illustration of convolving an example boxcar (Adapt 32 s condition) with exponential functions (leaky integrators). (**b**) Example of single-exponential modelling procedure: a single leaky integrator is convolved with the boxcar, and the resulting output gives a prediction for the time course of the VAE. (**c**) Example of multiple-exponential modelling procedure: two leaky integrators with longer and shorter trial-constants respectively are each convolved with the boxcar in turn, and the summed output of both mechanisms provides a prediction for the time course of the VAE. In all cases, the procedure is then repeated for the remaining boxcars/adaptation conditions. Model parameters are optimised via maximum likelihood estimation to minimise the prediction error during the test periods across all conditions simultaneously.

To further interrogate the mechanisms underlying the VAE, we fit the data using both exponential (leaky integrator) and power function models^24–26^. A leaky integrator predicts an exponential decay of the response characterised by two parameters: a trial-constant (τ) which determines the rate of change, with larger values giving a slower change, and a gain parameter which determines the overall response amplitude. Similarly, a power function predicts a power-law decay, characterised by a rate parameter (α) and a gain parameter. Power functions may approximate the summation of a series of correlated exponential functions^26–28^, and thus a power model may produce a similar output to a multiple-exponential model. A series of box-car models were constructed to model the adaptation and test periods for each condition, comprising 40, 80, 160, and 320 trials for the 32 s, 64 s, 128 s, and 256 s adaptation periods respectively, and a further 30 trials for the test period. Each of these boxcars were then convolved with the leaky integrator(s) or power function. We tested the ability of our models to predict the group average VAE estimates (Fig. 3b). A single-exponential model was constructed by convolving a single leaky integrator with the boxcars. This can be expanded to a multiple-exponential model by convolving separate short- and long-term leaky integrators with the boxcars, and summing the outputs. A power-law based model was constructed by convolving a power function with the boxcar. In all cases, the convolved output during the test periods provides a prediction of the VAE which can then be compared against the real group average VAE magnitudes. The model parameters were then optimised via maximum likelihood estimation to minimise this prediction error. An illustration of the modelling procedure for the single- and multiple-exponential models is shown in Fig. 4. Finally, to compare model goodness of fits, we calculated corrected Akaike Information Criterion (AICc)^29,30^ and Residual Standard Error (RSE) values for each model.

**Figure 5:**
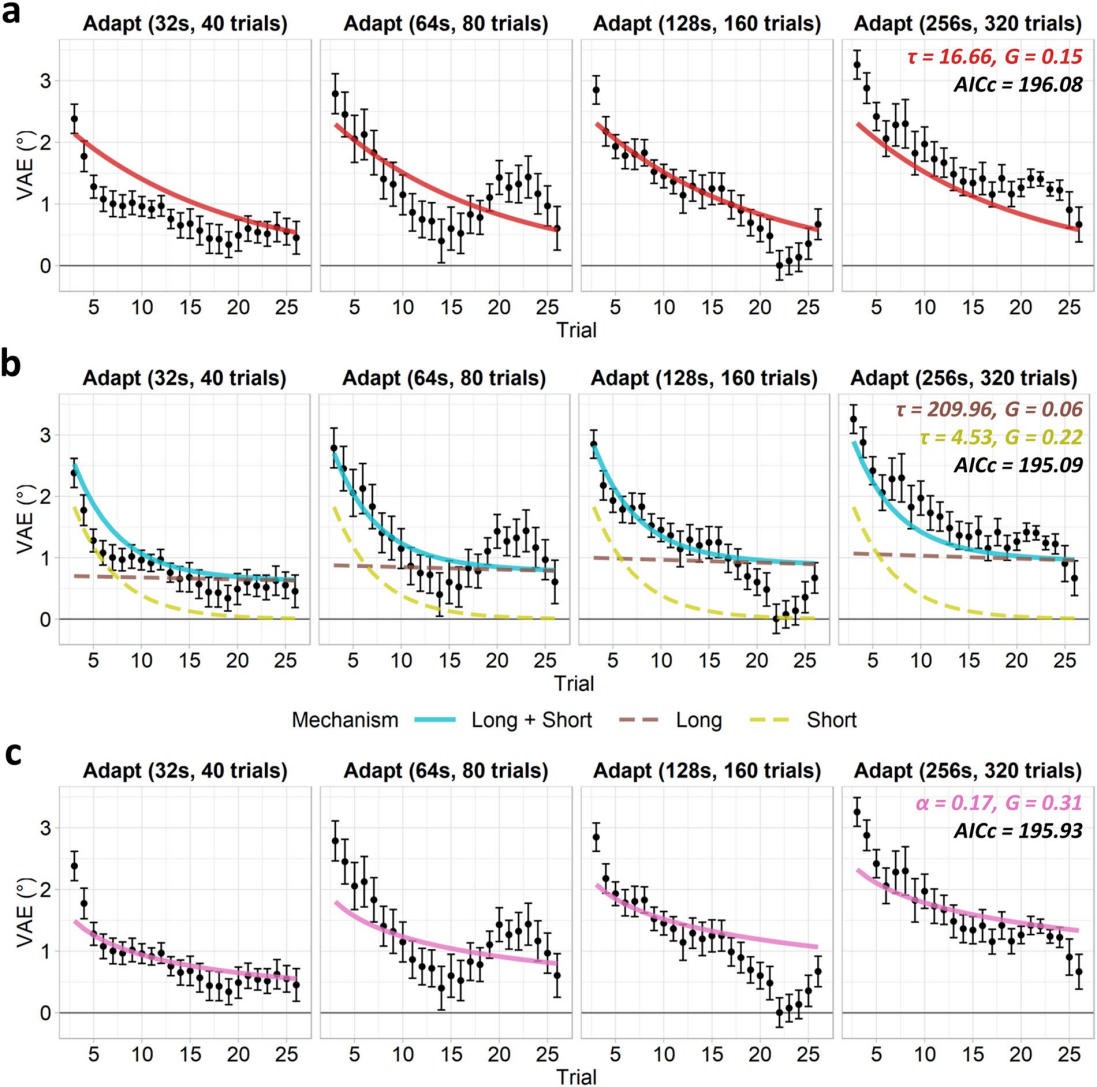
VAE magnitudes and model fits for standard adaptation conditions, excluding the adapt/de-adapt condition. Error bars indicate the standard error of the mean across subjects. Interpolated fits are shown for the (**a**) single-mechanism and (**b**) multiple-mechanism exponential (leaky integrator) models; annotations indicate best fitting trial-constant (τ) and gain (G) parameters. (**c**) Interpolated fit for power function model; annotations indicate best fitting rate (α) and gain (G) parameters. Corrected Akaike Information Criterion (AICc) values are also labelled.

We first tested the ability of these models to predict the responses in the standard adaptation conditions only, excluding the adapt/de-adapt condition (Fig. 5). A single-mechanism exponential model (Fig. 5a) provided an adequate fit to the data and suggested a relatively rapid decay of the VAE (τ = 16.66, gain = 0.15). Next, we fit a multiple-exponential model comprising two leaky integrator mechanisms, tuned to integrate information over longer and shorter time periods respectively (Fig. 5b). This model also provided a good fit to the data, with the short-term mechanism suggesting a rapid rate of decay (τ = 4.53, gain = 0.22), while the long-term mechanism suggested a considerably slower decay (τ = 209.87, gain = 0.06). The power model (Fig. 5c) also fit the data adequately (α = 0.17, gain = 0.31). AICc values revealed similar performance between the single-exponential (AICc = 196.08), multiple-exponential (AICc = 195.09), and power models (AICc = 195.93), and these values did not differ significantly (all pairwise *p* > 0.999). Residual standard errors were reduced for the multiple-exponential model (RSE = 0.33) compared to the single-exponential (RSE = 0.41) and power models (RSE = 0.40).

**Figure 6:**
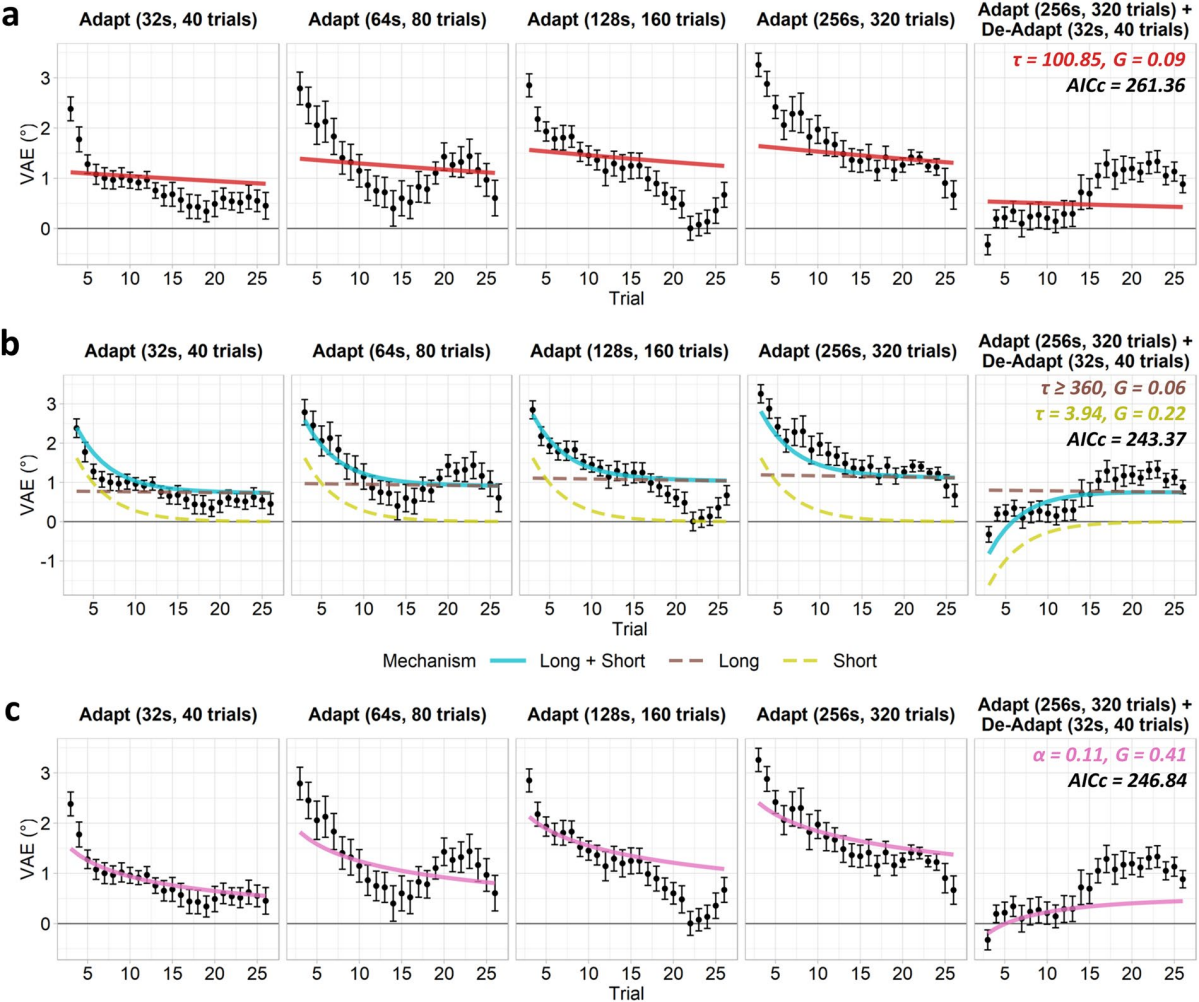
VAE magnitudes and model fits for all adaptation conditions, including the adapt/de-adapt condition. Error bars indicate the standard error of the mean across subjects. Interpolated fits are shown for the (**a**) single-mechanism and (**b**) multiple-mechanism exponential (leaky integrator) models; annotations indicate best fitting trial-constant (τ) and gain (G) parameters. (**c**) Interpolated fit for power function model; annotations indicate best fitting rate (α) and gain (G) parameters. Corrected Akaike Information Criterion (AICc) values are also labelled.

To better distinguish between the single- and distinct-mechanisms accounts, we next tested the ability of the leaky models to predict the VAE across all the adaptation conditions, including the adapt/de-adapt condition (Figs 2, 6). The single-mechanism exponential model now provided a poor fit to the data (Fig. 6a), with a slow rate of decay (τ = 100.84, gain = 0.09). A single exponential mechanism was thus unable to simultaneously capture the opposing effects of the adaptor and de-adaptor, and hence could not reproduce the delayed recovery of the VAE in the adapt/de-adapt condition. By comparison, the multiple-exponential model provided a good fit to the data (Fig. 6b) and again suggested a short-term mechanism with a rapid decay (τ = 3.94, gain = 0.22), and a long-term mechanism with a much slower decay that capped at the optimization routine’s upper bound for the trial-constant (τ ≥ 360, gain = 0.06). The short-term mechanism builds and decays rapidly and hence in the adapt/de-adapt condition is primarily driven by the more recent de-adaptor, whilst the long-term mechanism adapts more slowly and so is more heavily influenced by the longer duration adaptor. Consequently, multiple exponential mechanisms can simultaneously incorporate conflicting information across different timescales and so are able to capture the delayed recovery of the VAE. The power model (α = 0.11, gain = 0.41) struggled to fully capture the pattern of results across conditions. The model was able to partially reproduce the delayed recovery of the VAE in the adapt/de-adapt condition, but undershot the magnitude of the effect. AICc values revealed poorer fits in the single-exponential (AICc = 261.36) than the multiple-exponential (AICc = 243.37) or power models (AICc = 246.84). These values differed significantly between the single- and multiple-exponential models (*p* < 0.001), and between the single-exponential and power models (*p* = 0.001). Although visual inspection of the model fits (Fig. 5b,c) suggests better fits for the multiple-exponential over the power model, these AICc values did not differ significantly (*p* = 0.176) as the multiple-exponential model is penalised for its larger number of parameters. Nevertheless, residual standard errors were lower for the multiple-exponential (RSE = 0.35) than the single-exponential (RSE = 0.56) or power models (RSE = 0.43).

## Discussion

We have used the “ventriloquist aftereffect” (VAE) to quantify the dynamics of spatial multisensory recalibration and distinguish whether unitary or distinct mechanisms operate at different timescales. The VAE rapidly saturated but decayed exponentially, consistent with both transient and sustained adaptation. When long-term adaptation to a spatial offset was immediately followed by a brief period of de-adaptation to an opposing offset, VAEs initially cancelled each other but subsequently reappeared with further testing. These data were best fit with a multiple-exponential model that integrated information over both the recent and more remote past. Taken together, these findings suggest that multisensory adaptation is underpinned by distinct recalibration mechanisms that operate at different timescales.

Although a reliable VAE was observed in all conditions, neither the magnitude of the VAE nor the rate of its decay differed substantially with increasing durations of adaptation. This would suggest the VAE built and saturated quickly – potentially even within the period of our shortest adaptation condition of 32 seconds. Indeed, the short-term mechanism of our multiple-exponential model predicted a relatively short trial constant that yielded complete saturation within the shortest adaptation period. This is consistent with a previous study by Frissen and colleagues that suggested the VAE saturates following between 30 seconds and 1 minute of adaptation^12^. Similarly, spatial recalibration effects have been reported following very brief periods of adaptation, potentially down to just a single trial^16–18^.

Although the VAE built and decayed quickly, it often failed to completely decay to zero within the 30 trials of the testing run. The long-term mechanism of our multiple- exponential model predicted a much more gradual rate of change that yielded a more sustained response across the test period. Indeed, the trial-constant parameter (τ) capped at the upper bound set for the minimisation algorithm, suggesting a rate of decay occurring across a much greater timescale than was present within our testing runs. Testing for more extensive periods may help to better resolve the parameters of this longer-term mechanism. Consistent with these results, Frissen and colleagues also reported a longer-term sustain of the VAE^12^. This would support a distinct-mechanisms account, where short-term mechanisms sensitive to the immediate past yield a rapid build-up and decay of the VAE, whilst longer-term mechanisms with a much slower rate of decay yield a more sustained response in later time periods.

To more explicitly test the possibility of distinct mechanisms operating at different timescales, we employed an adapt/de-adapt paradigm^21,25^ in which participants first adapted to a given offset for an extended period (256 seconds) then immediately de-adapted to the opposing offset for a much shorter duration (32 seconds) before testing. As predicted, this paradigm resulted in vastly reduced VAEs in trials immediately after cessation of the de-adaptation period, when both long and short-term mechanisms remained active but in opposing directions and hence cancelled. However, as the effects of the short-term mechanism decayed rapidly whilst those of the longer-term mechanism sustained, a VAE was seen to re-emerge in later trials in the direction predicted by the initial adaptor. This demonstrates that VAEs with opposing directions but occurring across different timescales could be simultaneously maintained and suggests a model of the VAE in which distinct mechanisms adapt over different timescales. Consistent with this, our leaky integrator exponential models showed that whilst a single-mechanism could adequately predict the responses to the standard adaptation conditions alone, only multiple distinct mechanisms integrating information over both the recent and remote past were able to predict the responses in the adapt/de-adapt condition. Future research could further interrogate the temporal scales of these mechanisms using alternative adaptation paradigms. For instance, an adapt/de-adapt/re-adapt paradigm could be employed, in which a distinct-mechanisms model would predict a more rapid growth of the VAE during re-adaptation than the initial adaptation^21,25^.

We also tested the ability of a power function model to predict the VAE across conditions, as such models can approximate the summation of multiple correlated exponential models^26–28^, and a recent study of the VAE suggests approximately similar performance between a multiple-exponential and a power model^24^. This model struggled to fully capture the pattern of responses across our conditions. Although it did partially reproduce the delayed recovery of the VAE in the adapt/de-adapt condition, it nevertheless undershot the magnitude of the effect. The delayed recovery of the VAE in the adapt/de-adapt condition critically depends on integrating opposing sensory information across different timescales. Whilst our results generally favour an account in which separable neural mechanisms are tuned to different timescales (akin to a multiple-exponential model), it remains possible that these effects could be predicted by a single neural mechanism that nevertheless integrates information across distinct timescales simultaneously (akin to a power model). Importantly, however, both of these accounts remain consistent with the VAE being underpinned by distinct recent and remote recalibration mechanisms.

A distinct-mechanisms model is not without precedent. Previous studies of the VAE have identified effects of frequency-dependence^17^ and spatial reference frames^31^ varying with increasing adaptation duration, suggesting a change in adapting mechanisms. More generally, audio-visual recalibration effects have been reported following adaptation across a range of timescales, from just a few seconds to upwards of several minutes, and for several perceptual dimensions including spatial location^7–9,14,16–18^ and temporal synchrony^1,4–6,15^. Mechanisms operating at distinct timescales have also been proposed to account for various unimodal adaptation effects, from perception of relatively low-level features such as visual orientation^21,22^, up to much higher-level processes such as face perception^20^. This raises the possibility that adapting mechanisms operating at distinct timescales are a more ubiquitous property of sensory recalibration. Sensory changes may occur across a wide range of timescales, from relatively brief and transient changes such as those caused by an organism transitioning between different environments, to those that may span much longer periods such as developmental changes throughout childhood. Mechanisms sensitive to the more recent past have the potential to more flexibly recalibrate to brief environmental changes, but also risk being more subject to transient sources of sensory noise. By maintaining sensitivity to the temporal scale over which sensory changes occur, the perceptual system can remain optimally tuned to changes over a wide range of timescales, whilst at the same time balancing the flexibility of rapidly tuned mechanisms against the long-term reliability of more sustained mechanisms^19^.

One issue that remains unresolved is the extent to which the growth and decay of recalibration effects may be understood in terms of exact timescales (e.g. as measured in seconds) versus an accumulation of sensory evidence over time (but not necessarily linked to exact units of time). Studies in other domains have supported evidence-based accounts; for instance, storage of adaptation – in which aftereffects sustain for longer time periods in the absence of further sensory evidence ‒ has been reported in both unimodal (e.g. visual contrast adaptation^32^) and multimodal domains (e.g. audio-visual temporal recalibration^33^). However, the test phases in our experiment contained only unimodal auditory stimuli, and hence did not present further multimodal evidence of audio-visual spatial relationships. Consequently, it seems difficult to explain the decay of the VAE in our experiment purely by accumulation of sensory evidence. Thus, our experiment instead appears more in line with a timescale-based account. Nevertheless, an evidence-based account cannot be entirely dismissed. For instance, we measured the progression of the VAE in units of trials rather seconds, so our results are not purely explained in terms of exact timescales either.

An open question for a distinct-mechanisms account would be whether each mechanism relies on similar or distinct neural components. One possible candidate for the neural locus of the VAE is primary auditory cortex. Pairing visual information with an auditory signal modulates responses in primary auditory cortices^34^. The VAE itself correlates with responses in primary auditory cortices^35^, and is associated with early latency electrophysiological components^36^, suggesting a reliance on relatively early processing stages. The immediate spatial capture of sounds by vision (without adaptation) has again been associated with primary auditory cortices, but also later electrophysiological components^37^, suggesting a greater degree of mediation by higher-level processing stages. This suggests more immediate audio-visual recalibration may rely more on interactions between early sensory cortices and higher-level multisensory regions^38^, whilst more sustained adaptation effects may lead to a more permanent recalibration of early sensory cortices driven by top-down feedback. Different adapting mechanisms operating at different timescales may entail a shift in the neural locus and interactions between regions. Under this hypothesis, qualitatively different behavioural effects of multisensory recalibration may be expected across varying timescales. Indeed, different timescales of recalibration have been shown to affect both the frequency-dependence^17^ and spatial reference frames^31^ of the VAE.

In conclusion, we used the ventriloquist aftereffect to examine the mechanisms underpinning multisensory recalibration across differing timescales. Our results support an account in which distinct adapting mechanisms integrate information over different temporal scales. This enables perceptual systems to correct for inter-sensory discrepancies by optimally tuning into the timescales over which sensory changes occur in the environment.

## Methods

### Participants

21 participants (10 male, 11 female, median age = 22, age range = 20–46) took part in the first experiment. Data from one participant were excluded due to difficulties experienced in localising the sound sources and performing the localisation task. 18 participants (8 male, 10 female, median age = 22, age range = 20–46) took part in the second experiment; all participants in the second experiment also participated in the first. The study was approved by the ethics committee of the School of Psychology, University of Nottingham, and all procedures were conducted in accordance with the relevant guidelines and regulations of the committee, and in accordance with the Declaration of Helsinki. All participants gave informed written consent to participate in the study.

### Stimuli

Visual stimuli were projected onto a large semi-circular screen (radius = 2.5 m, height = 2 m ≈ 43.60° visual angle) that wrapped 180° in azimuth around the participant. Video feeds were projected by 3 interleaving projectors, and Immersaview’s Sol7 software (https://www.immersaview.com/) was used to blend the feeds and correct for the warp of the screen. Visual stimuli during the adaptation phases were 2-dimensional luminance Gaussian blobs (FWHM = 5° visual angle), presented across a range of azimuths but always at 0° elevation. These were presented for 500 ms and were sinusoidally contrast modulated (rate = 6 Hz, depth between 50% and 100% of maximum contrast). During test phases, a visual marker subtending 1° of visual angle and the full height of the screen was presented. A pair of vertical lines were presented throughout the entire experiment above and below fixation, at 0° azimuth and a sufficient vertical distance from 0° elevation so as not to occlude the Gaussian blobs. Participants were instructed to keep their head oriented straight ahead and to fixate in between the lines at all times. The colour of the lines also cued the current experiment phase: lines appeared red during adaptation phases and blue during test phases.

Audio stimuli were pink-noise bursts, presented binaurally over Sennheiser HD265 headphones. Stimulus azimuth was simulated using head-related transfer functions (HRTFs) from the MIT Kemar database^39^, which provides azimuths up to ±90° in 5° intervals. To encourage perceptual binding of visual and auditory stimuli, virtual reverberations were added to the auditory signals using the image-source method^40^ to simulate sources at the distance of the projection screen. The participant was modelled as sitting 2.7 m from the left and 1.5 m from the back of a 4.2 × 5.2 m sized room, corresponding to the dimensions of the testing room. Sources were emulated as originating from an arc (radius = 2.5 m) wrapping around the front of the participant, corresponding to the projection screen. Reverberations comprised up to 5 reflections and assumed walls with a uniform absorbance of 0.2. An impulse response was constructed by collating the predicted incoming pulses at the participant’s location following the reverberations. This was then convolved with the Kemar HRTFs to yield a new set of HRTFs that, when convolved with an input signal, would simulate both the source azimuth and reverberations according to source distance. The sound signals themselves were 500 ms pink-noise bursts (100–4000 Hz bandpass) which were sinusoidally amplitude modulated (rate = 6 Hz, depth = 3 dB). Signals then had onsets and offsets gated by 25 ms raised-cosine ramps, before finally being convolved with the HRTFs. Stimuli were sampled at 44.1 kHz, and the average listening level was measured to be 62 dB(A) SPL for the stimulus at 0° azimuth.

During adaptation phases, visual and audio stimuli were presented synchronously. Both stimuli were presented for 500 ms duration and with a 300 ms inter-stimulus interval. To encourage perceptual binding, visual and audio stimuli were sinusoidally modulated in synchrony. To facilitate allocation of spatial attention to the stimulus location, audio-visual pairs were presented 5 times consecutively at each location^8^. Audio-visual pairs were presented with either −20° (audio left of visual), 0°, or 20° (audio right of visual) offsets in azimuth; offsets always refer to the location of the audio relative to the visual stimulus. Spatial offsets were applied evenly on either side of the target location, e.g. a stimulus pair presented at 15° azimuth with a 20° spatial offset would comprise a visual stimulus at 5° and an audio stimulus at 25°. Target locations ranged between −35° (left) and +35° (right) azimuth in either 5° (experiment 1) or 10° (experiment 2) increments.

During test phases, audio stimuli (specifications same as for adaptation phase) were presented unimodally. Stimulus location was randomly selected on each trial from a normal distribution (μ = 0°, σ = 20°) between −35° (left) and 35° (right) azimuth in 5° steps. After each stimulus presentation participants were required to reproduce the perceived auditory location (azimuth). A visual marker was presented on screen which participants could move left and right with a mouse to indicate the stimulus location. Participants entered their response by mouse click, after which the next trial would be presented following an inter-trial interval of 200 ms.

All experiments were run using custom software written in Python (PsychoPy^41,42^, http://www.psychopy.org/).

### Experiment 1: magnitude of spatial recalibration

#### Procedure

In an initial experiment, we sought to replicate the basic VAE with our experimental paradigm. The experiment employed a blocked design, with each block presenting a particular adaptation spatial offset (−20°, 0°, 20°). Adaptation stimuli were presented between −35° (left) and 35° (right) azimuth in 5° increments (15 locations total). Each adaptation phase lasted 60 s, which comprised one full pass over all locations in a randomised order. Following each adaption phase, participants completed 10 test trials in which they reproduced the locations of unimodally presented auditory stimuli (see above). Each block comprised 5 adapt/test cycles in this manner, and participants completed 2 blocks per adaptation offset (6 blocks total). Across all blocks, participants therefore provided 100 responses per adaptation offset.

#### Statistical analyses

Stimulus azimuth was assigned as the predictor variable and perceived azimuth was assigned as the outcome variable. First, outliers were rejected for each adaptation offset and participant independently using a robust Mahalanobis distance metric^43^. For a given set of samples, the distance for each sample from the centre of the cluster was measured using Mahalanobis distance. To avoid the distance metric itself being biased by outliers, a minimum covariance determinant estimate^44^ was used to obtain the covariance matrix. Repeated sub-samples each comprising 75% of all samples were taken from the dataset and the determinant of the covariance matrix calculated for each one. Mahalanobis distances were based on the covariance matrix of the sub-sample with the lowest covariance determinant value as this is the least likely to include outliers. Distances were then converted to probabilities via a chi-square distribution, such that more distant samples were considered less probable. An alpha criterion of *p* < 0.01 was used to identify and reject outliers; on average this resulted in the rejection of approximately 3.93% of total trials.

Data were entered into mixed-effects linear regression analyses for each adaptation offset separately, allowing random intercepts and slopes across participants. Spatial bias was quantified by the intercept coefficients of the models, whilst spatial gain was quantified by the slope coefficients. To test for differences between the adaptation offset conditions, the mixed-effects coefficients for each parameter (spatial bias/intercepts, spatial gain/slopes) across participants were entered into a one-way repeated measures ANOVA with a main factor of adaptation offset (−20°, 0°, 20°). Greenhouse-Geisser corrections were applied where data violated the assumption of sphericity. Effect sizes are reported using eta-squared. Post-hoc paired-samples t-tests contrasted the pairwise combinations of adaptation offsets (−20° > 0°, 0° > 20°, −20° > 20°) subject to a Bonferroni-Holm correction for multiple comparisons^45^. Effect sizes are reported using Hedges’ *g*_*av*_, whereby the mean of the condition pairwise differences is standardised by the average of each condition’s standard deviation and then corrected for bias^46,47^. All statistical tests were two-tailed and employed an alpha criterion of 0.05 for determining statistical significance.

### Experiment 2: timescales of spatial recalibration

#### Procedure

Stimulus parameters and procedures of the second experiment are the same as the first, with the following exceptions. During the adaptation phase, stimuli were presented between ±35° azimuth in 10° intervals (8 locations total). We manipulated the duration of the adaptation phase, between 32, 64, 128, and 256 seconds, corresponding to 1, 2, 4, and 8 passes over all locations respectively. Locations were selected in a pseudo-random order across passes. In addition, we included an adapt/de-adapt condition in which participants adapted to a given spatial offset for 256 seconds, then immediately de-adapted to the opposing offset for 32 seconds. All of the standard adaptation conditions were repeated for spatial offsets of −20°, 0°, and 20°, whilst the adapt/de-adapt was repeated for the −20° and 20° offsets. This gave a total of 14 adaptation conditions across all offsets and durations, each of which was repeated twice across a total of 28 blocks. After each adaptation phase, participants completed 30 test trials, performing the reproduction task as described above. Each block comprised 2 adapt/test cycles in this manner.

#### Statistical analyses

Data were analysed using a sliding window of 7 trials incremented in 1 trial intervals. This window size provided a reasonable compromise between the greater temporal resolution of shorter windows and the higher signal-to-noise ratio of longer windows. This yielded 504 samples per window per condition (28 samples per participant per window per condition). Multivariate outlier rejection via robust Mahalanobis distance^43^ was performed (as described above) for each window, condition, and participant separately; on average this resulted in the rejection of approximately 7.32% of total trials. Data for a given window and condition were then entered into a mixed-effects linear regression analysis, allowing random intercepts and slopes across participants. The magnitude of the VAE was calculated by contrasting spatial bias (intercept) coefficients for the −20° over the 0° adaptation offset conditions, and the 0° over the 20° adaptation offset conditions, and then taking the trial-wise average of the two contrasts. There was no 0° adaptation offset for the adapt/de-adapt condition, so estimates were instead contrasted against the 0° adaptation offset estimates from the 256 seconds standard adaptation condition. In all cases, coefficients and VAE values were assigned to the middle trial of each window.

#### Modelling timescales of recalibration

Exponential (leaky integrator) and power functions were used to model the growth and decay of the VAE^25,26^. The leaky integrator takes the form *L* = *e*^−*τ*/*t*^, where τ is a trial-constant (analogous to a time-constant) that determines the rate of change (with larger values giving slower change), and *t* represents the trial number. The power function takes the form *P* = (*t* + *α*)^−1^, where α is a rate parameter and *t* represents the trial number. In both cases, the function outputs were normalised to sum to 1, then multiplied by a gain parameter that determined the amplitude of the response. For each adaptation duration, a simple boxcar model was constructed that predicted a VAE response of 20° during adaptation, −20° during de-adaptation (if applicable), and 0° during the test periods. The duration of the adaptation periods was based on the number of trials within those periods (40, 80, 160, and 320 trials for 32, 64, 128, and 256 seconds of adaptation, respectively), and the duration of the test period was always 30 trials. The leaky integrator/power function was convolved with the boxcar for each adaptation duration, and the output during test periods was used to predict the group average VAE estimates. Model parameters were optimised to minimise the prediction error via maximum likelihood estimation. For the leaky integrator, optimisation of the τ parameter was bounded below 360, which is the total number of trials in the combined adaptation and de-adaptation phases of the adapt/de-adapt condition. The error was minimised across all adaptation duration conditions simultaneously, such that a single set of parameters describes the output of the model across all conditions. Models were fit to the data both with and without inclusion of the adapt/de-adapt condition. An illustration of the modelling procedure is shown in Fig. 4.

For the single-mechanism exponential and power models, a single leaky integrator/power function was convolved with the boxcars and the output taken directly from this. In the multiple-exponential model, separate short- and long-term leaky integrators were defined and each convolved with the boxcars, and the outputs then summed together. The long-term mechanism was always constrained to have a longer trial-constant (τ) and smaller gain parameter than the short-term mechanism.

Goodness of fits between models were compared using corrected Akaike Information Criterion (AICc) values^29,30^ and Residual Standard Errors (RSEs). Smaller AICc values indicate a better goodness of fit to the data. The weight of evidence for one model over another can be defined as $p={e}^{(AIC{c}_{1}-AIC{c}_{2})/2}$, where *AICc*_1_ and *AICc*_2_ refer to the smaller and larger AICc values respectively. If this probability is less than the alpha criterion (α = 0.05) then this can be taken as sufficient evidence to reject the latter model in favour of the former. Fits were compared between all pairwise combinations of models (single-exponential, multiple-exponential, power) subject to a Bonferroni-Holm correction for multiple comparisons^45^.

## Data Availability

All materials, data, and stimulus presentation and analysis code are publicly available via the Open Science Framework and can be accessed at: https://osf.io/xktbz/.
